# Identification and analysis of divergent immune gene families within the Tasmanian devil genome

**DOI:** 10.1186/s12864-015-2206-9

**Published:** 2015-11-26

**Authors:** Katrina M. Morris, Yuanyuan Cheng, Wesley Warren, Anthony T. Papenfuss, Katherine Belov

**Affiliations:** Faculty of Veterinary Science, University of Sydney, Camperdown, NSW Australia; Washington University School of Medicine, 4444 Forest Park Ave, St Louis, MO 63108 USA; Bioinformatics Division, The Walter and Eliza Hall Institute for Medical Research, Parkville, VIC Australia; Bioinformatics and Cancer Genomics, Research Division, Peter MacCallum Cancer Centre, East Melbourne, VIC Australia

**Keywords:** Tasmanian devil, *Sarcophilus harrisii*, Conservation, Immunome, Cytokine, Chemokine Immunoglobulin, T-cell receptor

## Abstract

**Background:**

The Tasmanian devil (*Sarcophilus harrisii*) is being threatened with extinction in the wild by a disease known as devil facial tumour disease (DFTD). In order to prevent the spread of this disease a thorough understanding of the Tasmanian devil immune system and its response to the disease is required. In 2011 and 2012 two genome sequencing projects of the Tasmania devil were released. This has provided us with the raw data required to begin to investigate the Tasmanian devil immunome in depth. In this study we characterise immune gene families of the Tasmanian devil. We focus on immunoglobulins, T cell receptors and cytokine families.

**Results:**

We identify and describe 119 cytokines including 40 interleukins, 39 chemokines, 8 interferons, 18 tumour necrosis family cytokines and 14 additional cytokines. Constant regions for immunoglobulins and T cell receptors were also identified. The repertoire of genes in these families was similar to the opossum, however devil specific duplications were seen and orthologs to eutherian genes not previously identified in any marsupial were also identified.

**Conclusions:**

By using multiple data sources as well as targeted search methods, highly divergent genes across the Tasmanian devil immune system were identified and characterised. This understanding will allow for the development of devil specific assays and reagents and allow for future studies into the immune response of the Tasmanian devil immune system to DFTD.

**Electronic supplementary material:**

The online version of this article (doi:10.1186/s12864-015-2206-9) contains supplementary material, which is available to authorized users.

## Background

The Tasmanian devil (*Sarcophilus harrisii*) is the world’s largest surviving marsupial carnivore. The devil facial tumour disease (DFTD) is currently threatening Tasmanian devils with extinction. Since its emergence around 1996, the disease has spread rapidly across the state resulting in a population decline of around 80 % [1]. This had led to Tasmanian devils being listed as endangered by the International Union for Conservation of Nature (IUCN) [2]. An unusual feature of DFTD is that it is transmitted as an allograft when Tasmanian devils bite each other [3, 4]. This makes DFTD one of only three naturally occurring clonally transmissible tumours along with canine transmissible venereal tumour in dogs [5], and a recently identified transmissible cancer in clams [6]. This tumour is able to transmit between unrelated hosts without eliciting an immune response [7, 8], but how this tumour avoids the host immune system is not fully understood. The tumour down-regulates cell surface Major Histocompatibility Complex (MHC), which allows the tumour to ‘hide’ from the host immune system [9]. However, these observations do not fully account for the ability of DFTD to avoid immune recognition, as a lack of MHC expression should still elicit a NK cell response [10]. In the past, investigation into the immunology of the Tasmanian devil and its immune response to the tumour has been hampered by a lack of genomic and immunological resources. Characterisation of immune gene repertoires is important for the development of specific immune reagents necessary for vaccine development. In addition, gene identification and specific reagents are required for devil specific assays such as qPCR, immunohistochemistry, immunocytochemistry and flow cytometry. Since 2012 two Tasmanian devil genomes have been available [11, 12], one of which is available on the Ensembl genome browser. While many Tasmanian devil genes have been annotated in the Ensembl pipeline, numerous divergent genes of the immune system were missed by the Ensembl annotation or have been poorly annotated. Several families of the Tasmanian devil immune system have been characterised in previous studies, including natural killer cell receptors [13], major histocompatibility complex (MHC; [14, 15]) and toll-like receptors (TLR; [16]). Several additional divergent immune gene families, important for characterisation of immune response, have not been investigated in the Tasmanian devil, including cytokine families, immunoglobulins and T-cell receptors. Within the marsupials these families have been best characterised in the gray short-tailed opossum (*Monodelphis domestica*), for which a good quality genome assembly is available [17]. Some genes have also been characterised in additional marsupials including tammar wallaby (*Macropus eugenii*), brushtail possum (*Trichosurus vulpecula*) and koala (*Phascolarctos cinereus*) [18–21]. Research into the marsupials thus far has demonstrated that the marsupial immunome is similar to that of eutherian species, although notable differences occur including marsupial specific expansions within chemokines [22], the absence of IgD [19] and the presence of a T cell receptor found only in marsupials and monotremes [19].

Cytokines are a diverse group of secreted proteins produced by a broad range of cells that act as mediators of the immune system. Different cytokine profiles are associated with Th1 and Th2 immune responses, and thus can be used to characterise the immune response to pathogens [23]. Groups of cytokines include chemokines, interleukins, interferons, tumour necrosis factors (TNF) and growth factors. Interleukins are a diverse group of cytokines; in humans there are 36 interleukins with roles in cell proliferation, maturation, migration and adhesion [24], while 34 interleukins have been identified in opossum [22]. Chemokines are involved in inflammation, cell migration, activation and differentiation [25]. Within the chemokines are four subclasses defined by their characteristic spacing of cysteine residues (C, CC, CXC and, CX3C). The chemokine family is a dynamically evolving family, with some chemokines being conserved among vertebrates, while lineage specific duplications of chemokines are seen particularly within the CC and CXC subclasses [22]. Interferons are related group of cytokines with a critical role in anti-viral immune response [26]. Within the interferons are three subclasses. Type I contains several members including IFN-α - β and -κ while the Type II interferon class has a single member (IFN-γ). In the opossum seven IFN-α genes have been identified, while single - β, - κ and -γ are present [22]. Type III interferons are known as IFN-λ interferons. Tumour necrosis factors are a family of cytokines that can elicit apoptosis of cells and in humans, 19 members of this family have been recognised [27].

There are two antigen receptor systems in vertebrates that allow for antigen specific binding: immunoglobulins (Ig) and T cell receptors (TCR). Immunoglobulins, produced by B cells, are formed by two identical heavy and light chains with each chain consisting of a constant and a variable region [28]. Multiple heavy chain constant regions are encoded in the genome and these determine the isotype of the immunoglobulin. There are five Ig isotypes present in mammals, which are defined by their heavy chain constant region (A, D, G, E, M) and there are two light chain classes seen in vertebrates (λ and κ) [28]. In the marsupials single A, G, E and M regions have been identified, as well as single κ and multiple λ domains, while an ortholog to D has not been identified [29].

T cell receptors allow for recognition of MHC bound antigens. These receptors are made of two TCR chains. Like Igs, TCR contain variable regions and constant regions which define the chain class. These chains include α, β, δ and γ in all studied mammalian species; these classes may contain single or multiple constant domain loci [19]. Within the marsupials and monotremes exists an additional TCR chain, μ, which in studied species has multiple constant domain loci [30].

Little is known about these genes in marsupials outside of the opossum. However, the opossum, an American marsupial, diverged from Australian marsupials about 80 million years ago [31]. Therefore we expect to see differences in the immune gene repertoires of these two species. In this study we identify and characterise these divergent immune gene families within the Tasmanian devil genome assembly. To aid in immune gene identification and characterisation we have produced transcriptomes for Tasmanian devil lymph and spleen.

## Results and discussion

We have used multiple data sources and a combination of search methods to locate divergent Tasmanian devil immune genes. The data sources include two genomes [11, 12] and five transcriptomes (testis, tumour [32], milk, spleen and lymph; see Additional file 1 for accession numbers). Search methods utilised included basic local alignment search tool (BLAST) searches, hidden Markov model (HMMER) searches and searches based on conserved synteny. We report the identification of 141 Tasmanian devil immune genes including cytokines, chemokines, Igs and TCRs. All identified sequences have been deposited into a publicly available database (http://hp580.angis.org.au/tagbase/gutentag/).

### Interleukins

**Fig. 1:**
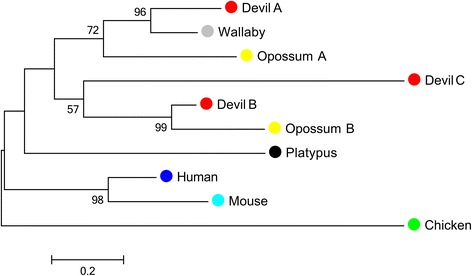
Phylogeny of IL18 amino acid sequences. Maximum likelihood tree of IL18 amino acid sequence with 1000 bootstrap resamplings, including devil (*red*), wallaby (*gray*), opossum (*yellow*), platypus (*black*), human (*blue*), mouse (*cyan*) and chicken (*green*) sequences. Bootstrap value greater than 50 % only are displayed. There are three sequences (Devil A, B and C) in the Tasmanian devil and two (Opossum A and B) in the opossum due to duplications in the genome of these species

A total of 40 interleukin genes were predicted from the Tasmanian devil genome (Table 1; Additional file 2). Of these 28 were orthologs of human interleukins while the remaining 11 represented marsupial or devil specific duplications. These included three homologs of human *IL18* and two genes related to the IL-36 family of genes in eutherian mammals. *IL18* is highly conserved among most vertebrate lineages, being present as a single copy in species of fish, birds, reptiles and mammals [33–35]. Although only a single *IL18* ortholog was identified in a previous study on opossums [22] an additional *IL18* homolog was identified by the Ensembl annotation [Ensembl:ENSMODG00000014381] adjacent to the previously identified *IL18*. To our knowledge duplication of the *IL18* locus has not been previously reported in any species. To explore the evolutionary relationships between mammalian IL-18 sequences a phylogeny was constructed (Fig. 1). The single identified wallaby IL-18 forms an orthologous relationship to one of the devil and opossum IL-18 sequences. The second opossum sequence appears to be orthologous to a second devil sequence, while the third devil sequence has no clear orthologue. This indicates that the duplication of *IL18* may have occurred early on marsupial evolution rather than representing lineage specific duplications within marsupial lineages. Only one of the copies of *IL18* was expressed in the available Tasmanian devil transcriptomes (*IL18A*; expressed in testis, tumour and milk transcriptomes; Additional file 3) so it has yet to be seen whether the additional copies are expressed.

**Table 1:** Summary table of cytokine, immunoglobulin and T-cell receptor sequences identified in Tasmanian devil

Interleukins	Chemokines	TNF family	Ig constant regions
IL10	CC family	TNF	A
IL11	CCL26	LTA	G
IL12A	CCLD1^a^	LTB	E
IL12B	CCLD2^a^	TNFSF4	M
IL13	CCLD3^a^	CD40LG	K
IL15	CCLD4^a^	FASLG	L1^a^
IL16	CCLD5^a^	CD70	L2^a^
IL17A	CCLD6^a^	TNFSF8	L3^a^
IL17B	CCLD7^a^	TNFSF9	L4^a^
IL17C	CCLD8^a^	TNFSF10	
IL17D	CCLD9^a^	TNFSF10L	TCR constant regions
IL17F	CCLD10^a^	TNFSF11	TRGC
IL18A^a^	CCLD11^a^	TNFSF13L^a^	TRAC
IL18B^a^	CCLD12^a^	TNFSF13B	TRDC
IL18C^a^	CCLD13^a^	TNFSF14	TRBC1^a^
IL19	CCLD14^a^	TNFSF15	TRBC2^a^
IL1A	CCLD15^a^	TNFSF18	TRBC3^a^
IL1B	CCLD16^a^	EDA	TRMC1^a^
IL1F10	CCL17		TRMC2^a^
IL1RN	CCL19	Interferons	TRMC3^a^
IL2	CCL20	IFNA1^a^	TRMC4^a^
IL20	CCL21	IFNA2^a^	TRMC5^a^
IL21	CCL22	IFNA3^a^	TRMC6^a^
IL22	CCL25	IFNA4^a^	TRMC7^a^
IL22F1^a^	CCL27	IFNB	
IL22F2^a^	CCL28	IFNG	
IL22F3^a^	CCL24	IFNL1^a^	
IL22F4^a^	CXC family	IFNL2^a^	
IL22F5^a^	CXCLD1^a^		
IL23A	CXCLD2^a^	Additional Cytokines	
IL24	CXCL8	CNTF	
IL25	CXCL9	CSF1	
IL26	CXCL10LA^a^	CSF2	
IL27	CXCL10LB^a^	CSF3	
IL31	CXCL11	CTF1	
IL33	CXCL12	KITLG	
IL36L1^a^	CXCL13L^a^	LIF	
IL36L2^a^	CXCL14	OSM	
IL36RN	CXCL16	SLC11A1	
IL4	CXCL17	SPP1	
IL5	XC family	TGFB1	
IL6	XCLA^a^	TGFB2	
IL7	XCLB^a^	TGFB3	
IL8	CX3C family	VEGFA	
IL9	CX3CL1		

^a^indicates a gene which is not a direct ortholog of a eutherian gene

A full length *IL22* gene was identified in the Tasmanian devil genome. This gene had five exons, as in the human *IL22*. However, an additional five partial genes or gene fragments were identified, representing at least three unique *IL22*-like loci. An alignment of all the Tasmanian devil IL-22 sequences identified is shown in Additional file 4. These gene fragments were all identified in short or highly fragmented contigs, therefore these fragments may be part of full *IL22* sequences. Some of the fragments identified could potentially be part of the same gene, for example the sequences *IL22F3*, - *F4*, and *-F5* could combine to encode a full *IL22* sequence. One of the *IL22* sequences (*IL22F1*) had no introns, and therefore is likely to be a processed pseudogene. A processed pseudogene is produced from the reverse transcription of an mRNA transcript with subsequent reintegration into chromosomal DNA [36]. Interestingly, this sequence shows higher identity to the *IL22F3* and *IL22F4* fragments (both sequences with introns), than the *IL22* sequence. Therefore the *IL22F1* sequence more likely was generated from the transcript of one (or both) of these sequences than from the *IL22* sequence. This provides further evidence that there is more than one full length *IL22* gene in the Tasmanian devil genome. In most species, including opossum, *IL22* is represented by a single sequence [22]. However, a duplication at the locus is seen in some strains of mice [37]. None of the Tasmanian devil *IL22* sequences were identified in the devil transcriptomes. Further investigation will be required to determine whether the Tasmanian devil may have more than one functional *IL22* gene.

Orthologs of the genes *IL3*, *IL32* and *IL37* were not identified. Orthologs to *IL32* and *IL37* have not, to our knowledge, been identified to any animal outside of eutherian mammals, including both opossum and wallaby [22]. Therefore it is likely that these two interleukins are specific to eutherian mammals. As in the opossum [22], *IL3* could not be identified in the Tasmanian devil genome, despite being present in both eutherian mammals and chicken. The syntenic region in the Tasmanian devil genome was searched, but as in opossum, this region was fragmented, so it is possible that this gene could not be identified due to this fragmentation. In total 13 of the 40 interleukin sequences were identified in one or more Tasmanian devil transcriptomes, with *IL16* being the most ubiquitously expressed, being expressed in all five transcriptomes (Additional file 3).

### Chemokines

**Fig. 2:**
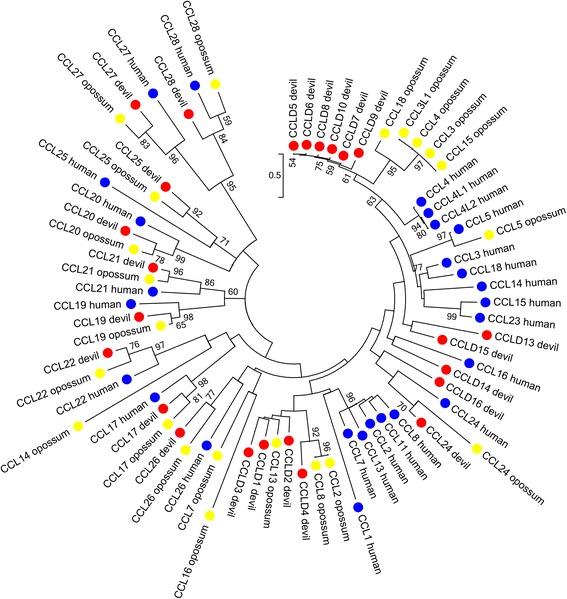
Phylogeny of CC family chemokine amino acid sequences from devil, opossum and human. Maximum likelihood tree with 1000 bootstrap resamplings. Bootstrap value greater than 50 % only are displayed. Devil, opossum and human sequences are indicated by red, yellow and blue dots respectively

Thirty-nine chemokines were identified in the Tasmanian devil genome (Table 1; Additional file 5). This is greater than that identified in the opossum (36) and the chicken (24), but less than the human (47) [22, 38]. Twenty-four chemokines of the CC family were identified, including orthologs to ten eutherian CCL chemokines (Fig. 2). Genes *CCLD5-CCLD10* appear to form a devil specific expansion (Fig. 2), and are likely to have duplicated recently due to their high identity (93.4–97.8 % amino acid identity) and their proximity to each other in the Tasmanian devil genome (See Additional file 5). These sequences lack direct orthology even to opossum sequences, and appear to be related to the human *CCL4* family. In addition, two pseudogenes related to this group were also identified in the Tasmanian devil genome, with either frame shifts or early stop codons. One CCL chemokine not identified, despite being identified in the opossum genome was *CCL5*. This gene could not be located by BLAST or HMMER searches in either the genome or in the transcriptomes. The genomic region syntenic to the opossum region containing *CCL5* was fragmented in the Tasmanian devil genome which may explain why this gene cannot be located. *CCL16* was not identified in devil or opossum, and this gene appears to be present only in the eutherian lineage. *CCL6*, *9*, and *12* were also not found, but these genes have thus far only been identified in rodents. A large number of CCL chemokines, in total thirteen, were identified in the transcriptomes (See Additional file 3). *CCLD14*, a chemokine with no identified orthologue in opossum or eutherians, was the most ubiquitously expressed being identified in all five of the transcriptomes.

**Fig. 3:**
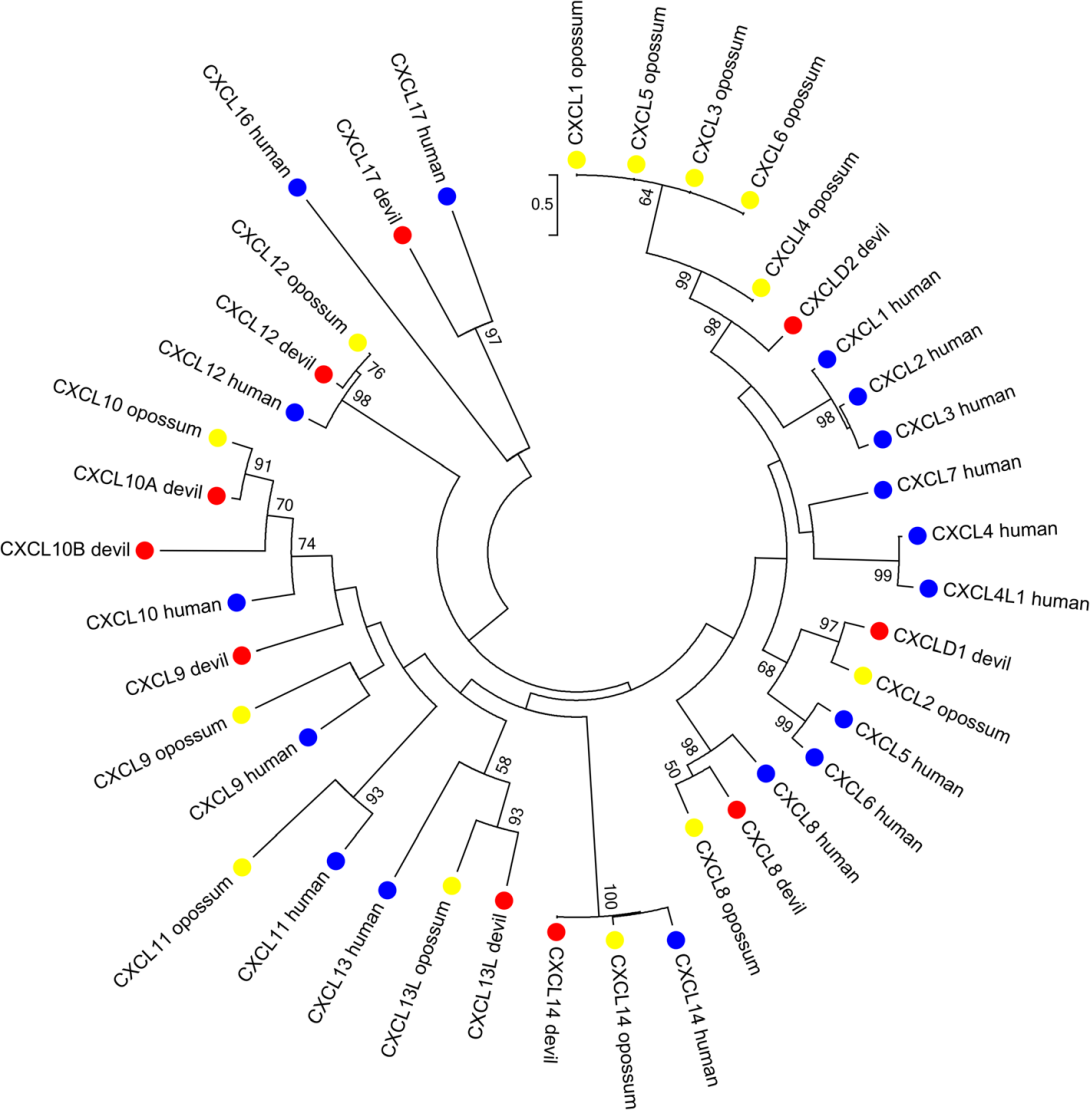
Phylogeny of CXC family chemokine amino acid sequences from devil, opossum and human. Maximum likelihood tree with 1000 bootstrap resamplings. Bootstrap value greater than 50 % only are displayed. Devil, opossum and human sequences are indicated by red, yellow and blue dots respectively

Twelve CXC chemokines were identified in the Tasmanian devil genome, nine of which were identified in the devil transcriptomes (Additional file 3). Eight of these are orthologs of eutherian chemokines (Fig. 3). Interestingly, while *CXCL10* is represented by a single gene in all species to our knowledge, two *CXCL10* homologs were identified in the Tasmanian devil genome, located adjacent to one another in the devil genome. While *CXCL10A* appears to be an ortholog to the opossum CXCL10 with strong bootstrap support, *CXCL10B* is more divergent (Fig. 3). The two Tasmanian devil genes were quite divergent (only 38 % amino acid identity) but both retained conserved CXC family residues including the cysteine residues, and so it is possible that both are functional. Only *CXCL10A* however, was expressed in a transcriptome (milk) so it is yet to be seen whether *CXCL10B* is also transcribed.

As in opossum and human, two XC chemokines are present in the Tasmanian devil genome, but these are not orthologous to either the human chemokines or the opossum (Additional file 6). Therefore, the duplication of an ancestral XCL gene likely occurred independently in the three lineages. The CX3C chemokine family is represented by a single gene in all species studied, and a single ortholog was identified in the Tasmanian devil genome.

### Interferons

**Fig. 4:**
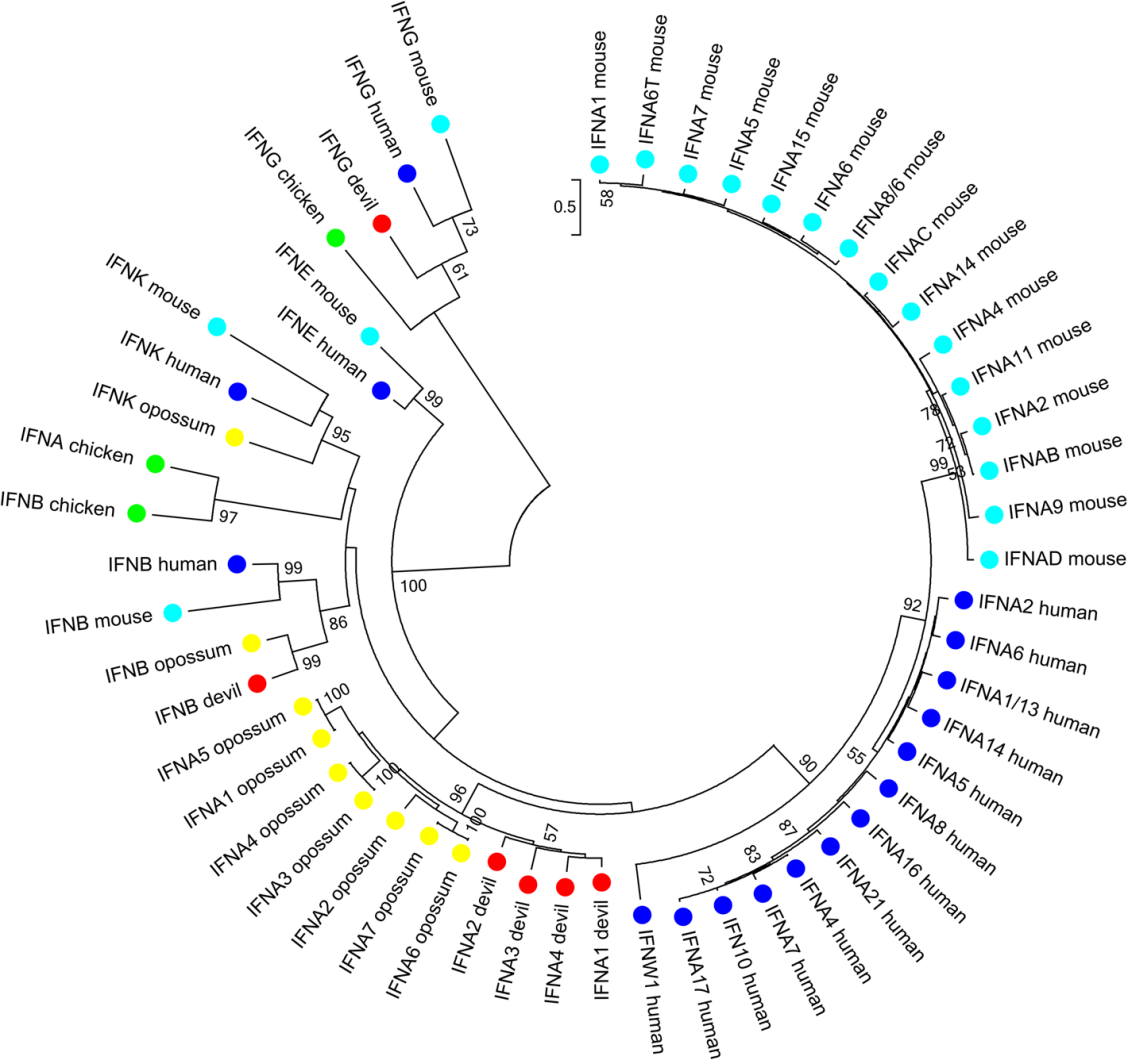
Phylogeny of interferon amino acid sequences from devil, opossum, human, mouse and chicken. Maximum likelihood tree with 1000 bootstrap resamplings. Bootstrap value greater than 50 % only are displayed. Devil, opossum, human, mouse and chicken sequences are indicated by red, yellow, blue, cyan and green dots respectively. Mammalian *IFNB*, *IFNG* and *IFNK* form single clades

Type I interferons in mammals include α, β, δ, ε, ω and κ interferons. In the opossum only α, β and κ type interferons have been identified [19]. In the Tasmanian devil an ortholog to the human and opossum *IFNB* was identified (Table 1; Additional file 7; Fig. 4). Mammalian species typically have multiple *IFNA* loci; humans have thirteen while in the opossum seven have been identified [22]. Only four *IFNA*s could be identified in the Tasmanian devil genome. However in addition, two *IFNA* pseudogenes were identified with early stop codons. These may represent recent loss of α interferon sequences, partly accounting for the difference in number between opossum and devil. In a phylogeny of interferons including human, mouse, devil and opossum (Fig. 4) *IFNA* genes formed species specific clades, suggesting that these genes evolve rapidly in both eutherian and marsupial species. *IFNK* could not be identified in either Tasmanian devil genome assembly, nor could it be identified in transcriptomes. The genes that occur upstream and downstream of *IFNK* in the opossum were identified in the Tasmanian devil genome, but these were at the ends of supercontigs, so it is likely that this gene is missing from the devil genome assembly. As in other marsupials, sequences encoding interferons δ, ε and ω were not identified in the Tasmanian devil genome.

Type II interferons are represented by a single gene in most species, *IFNG*. An ortholog to this gene was identified in the Tasmanian devil genome. The type III interferons group consists of IFN-λ molecules, in humans these are encoded by *IFNL1-3*, also known as *IL29*, *IL28A* and *IL28B*. Two *IFNL* genes were identified in the Tasmanian devil genome. These were not orthologous to the eutherian *IL28A/B* or *IL29* genes, but appear to be a marsupial specific duplication (Fig. 4). No interferon was expressed in any of the Tasmanian devil transcriptomes (Additional file 3).

### Tumour necrosis factors

Eighteen TNF family members were identified in the Tasmanian devil genome (Table 1; Additional file 8) nine of which were expressed in a devil transcriptome. Orthologs to most human members of the TNF family were identified in the Tasmanian devil genome. A single sequence (named *TNSF13L*) that showed homology to both *TNFSF12* and *TNFSF13* was identified in the Tasmanian devil. *TNFSF18* has not been previously predicted in any marsupials, though is present in both eutherians and platypus. Devil *TNFSF18* could not be identified using BLAST or HMMER searches, but was identified based on the conserved synteny of the region. *TNFSF18* was highly divergent from eutherian orthologs, with only 17 % amino acid identity between Tasmanian devil and human sequences, accounting for the difficulty in predicting this gene. *TNFSF10L*, a gene present in fish, birds and monotremes, but not in eutherian mammals, was also identified in the Tasmanian devil genome. In opossum, the *FASLG* sequence contained a frame shift mutation and its receptor *FAS* could not be identified, despite using the same search strategies used in the current study, leading to speculation that this ligand and receptor may not be functional in the opossum [22]. However, *FASLG* was identified in the Tasmanian devil genome without any features to suggest that it is a pseudogene and *FAS* was also identified. In addition both *FASLG* and *FAS* were expressed in the Tasmanian devil milk transcriptome, thus these proteins are likely to be functional in the devil.

Two TNF family genes (*TNFSF10* and *TNFSF11*) were difficult to predict in the Tasmanian devil genome due to exons being present on more than one scaffold, and being only partly present in either of the two genome assemblies. This indicates that the regions containing these genes may contain repetitive elements making them difficult to assemble. This also highlighted errors in the Ensembl 7.1 assembly; while the majority of the *TNFSF11* sequence was identified on a scaffold GL849887.1 flanked by two other genes, the first half of exon 1 was located on scaffold GL864876.1, within the intron of another gene. In addition the last exon was located on a third scaffold, GL851272.1. This arrangement is highly unlikely to represent the actual genomic arrangement and is likely the result of an error in the assembly.

### Additional cytokines

Fourteen additional cytokines not belonging to one of the above families were searched for, including the colony-stimulating factors and the transforming growth factor families (Table 1; Additional file 9). Orthologs to all of these genes were located in the Tasmanian devil genome, including *CNTF* which has not been previously predicted in marsupials. Several of these were highly divergent from their human and even opossum orthologs, including *CSF2* and *OSM*, and these were located using a combination of HMMER searches, synteny searches and FGENESH+ to predict the genes in the locations likely to contain the genes. *OSM* was one of the most divergent cytokines found in the Tasmanian devil with only 19 % peptide identity to the human sequence and 23 % to the predicted sequence in opossum.

### Immunoglobulins constant regions

In the Tasmanian devil genome A, E, G and M heavy chain constant regions were identified (Table 1; Additional file 10). As with the other marsupial species, such as wallaby and possum, that have been studied, the D locus could not be identified [19]. It is likely that this region has been lost in all marsupials. While G and A are often represented by multiple constant domain loci in eutherian mammals, as in other marsupials studied [19], these are only represented by single loci in the Tasmanian devil. The A, E and G loci were located adjacent to one another on a single scaffold while M was located on another scaffold. All four heavy chain loci were expressed in both the lymph and milk transcriptomes.

As in most other species the κ light chain is represented by a single locus in the Tasmanian devil genome. Four λ constant chains were identified compared to eight in the opossum [29]. The K and L loci were all located on separate scaffolds in the Tasmanian devil genome, indicating that this region is highly fragmented. Due to this it is possible that there may be additional L loci in the Tasmanian devil genome that could not be identified due to fragmentation. The κ and a single λ chain (λ4) were expressed in both the lymph and milk transcriptomes (Additional file 3). Therefore it is possible that in the Tasmanian devil light chain expression is dominated by single κ and λ light chains.

### T cell receptors

T cell receptor constant domains were also identified in the Tasmanian devil genome (Table 1; Additional file 11). Within most vertebrate mammals there are four TCR constant domains; α and δ which are typically found at the same locus, and β and γ which form unique genomic loci [19]. These are commonly represented by a single constant domain, except for β which is usually duplicated. Marsupials and monotremes have an additional TCR locus, the M locus, which contains multiple μ constant domains [19]. Constant domains from these five loci were identified in the Tasmanian devil genome. As with other mammals, the α and δ constant domains were found in close proximity, on contig GL834496.1. Three β domains were identified, compared to four which have been identified in opossum [39]. The single γ constant domain was also identified. A total of six μ constant domains were identified compared to eight in the opossum [39]. However, these were spread across five contigs in the Tasmanian devil genome, with most of these contigs being short and/or fragmented, so it is possible that additional domains exist that could not be identified due to fragmentation of the genome assembly. Heterodimers are formed by the α and β chains, and the δ and γ chains; in humans 95 % of TCR expression is composed of the α/β heterodimer [40]. Within the lymph node transcriptome α and β transcripts (*TRBC1*) were expressed, but not δ and γ, indicating that the α/β heterodimer may also dominate in the Tasmanian devil. Within the milk transcriptome *TRGC* and an additional β (*TRBC2*) were also identified. In addition μ chains were identified in both the lymph (*TRMC4*) and milk (*TRMC4*, *TRMC2*, *TRMC6*) transcriptomes. While variable domains for both the Igs and TCRs were identified, due to the highly fragmented nature of these regions it is highly likely that a large number of variable domains are missing from the genome and determining the genomic arrangement is not possible with the current genome assembly. With additional work to improve assembly of these regions in the Tasmanian devil genome this will be possible in the future.

### Genome limitations

A recurring problem with gene identification was the fragmentation of the genome assembly in the regions containing genes of interest. In some cases only partial genes could be predicted, with the predicted location of exons being beyond the end of the scaffold or in a break in the scaffold. Additionally, entire genes could not be located, but their predicted location was between two genomic scaffolds. Within highly duplicated groups of genes that are predicted to occur in the same genomic locus, such as the *TRMC* genes or *CCL* genes, fragmentation of these regions means that family members may be missing from the genome assemblies, and due to this we cannot definitively determine the number of sequences within these families in this study. While fragmentation was also an issue in identifying sequences in the opossum genome [22], the opossum genome generally has better coverage in these regions. For example the majority of *CCL* genes in the opossum could be identified in a single scaffold [22], while the majority of these in Tasmanian devil were found individually on unique scaffolds. This fragmentation makes examining genomic synteny on a wide scale difficult. Despite the issues with fragmentation, the vast majority of genes searched for in the Tasmanian devil genome were identified in this study. Additionally, access to two genome assemblies was beneficial for identifying genes in this study. The Murchison et al. [12] assembly, present on Ensembl, was the main genome used for gene identification as the scaffold length is generally much higher. However, genes missing, or partially missing from this assembly were located in the Miller et al. [11] assembly, including *CXCL16*, *TNFSF10*, *TNFSF11*.

## Conclusions

The availability of genomic and transcriptomic data has enabled us to investigate a broad range of immune genes for the first time in an Australian marsupial. Through the use of genomes and transcriptomes, diverse genes encoding critical components of the immune response can be identified and characterised, paving the way for future research into the immunology and diversity of the species. This is expected to be an increasingly important approach for research and conservation of threatened and endangered species in the future.

In this study a total of 141 immune genes were identified in the Tasmanian devil genome. While many of these were annotated in the Ensembl pipeline, 30 % of the genes searched for were either missed by the Ensembl annotation, or were poorly or partially annotated, particularly genes that are highly divergent from marsupial and eutherian orthologs. By using multiple data sources as well as targeted search methods, highly divergent genes were identified. In general, orthologs that were expected to be present in the Tasmanian devil genome were identified. Several eutherian genes that have not been previously identified in marsupials were also not identified in the Tasmanian devil, providing further evidence that these genes are likely to have either evolved within the eutherian lineage (including *CCL6*, *CCL16*, *IL32* and *IL37*) or have been deleted in the marsupial lineage (such as the IgD locus). A small number of genes that were expected to be present in the Tasmanian devil genome, including *IFNK* and *CCL5*, could not be identified by any method. The predicted locations of these genes are in fragmented regions of the genome assembly, and their absence from the transcriptomes is not unexpected. Therefore, it is more likely that these are missing from the genome assembly and not expressed in the transcriptomes, rather than being absent from the Tasmanian devil genome. Several genes that have not been previously identified in any marsupial species were identified in this study including *IL23A*, *TNFSF18* and *CNTF*.

Genes that formed devil or marsupial specific expansions were identified in the CXC and CC chemokine families and in the interferon α family. Expansions in these families have been previously seen in the opossum [19]. In the CC family, five CCL genes represent a lineage specific expansion in the devil. In addition, several unexpected duplications were seen in the Tasmanian devil genome. This included duplication of the *IL18*, *IL22* and *CXCL10* genes. In the future it would be interesting to determine whether more than one of these duplicated loci is functional.

Discovery of these genes is the essential first step to many research projects in the Tasmanian devil. This includes the development of antibodies, such as anti-devil IgG or anti-devil CD8, which allow for detailed immunological research of the devil and of DFTD, and for vaccine development [8, 41]. Identification of devil gene sequences are the first step for development of assays including qPCR, ELISA, immunohistochemistry and flow cytometry. These assays are crucial for investigating disease pathology and immune response in Tasmanian devils [8, 42, 43]. Genes described in this paper can be used to develop antibodies against Th1 and Th2 cytokines to further characterise devils’ response to DFTD. In addition, discovery of these genes is essential for looking at genetic variation at these immune genes that could correlate to DFTD resistance and could vary across populations in the wild. Tasmanian devils have a lack of genetic diversity which is believed to have made the Tasmanian devil susceptible to disease outbreaks [14, 44]. Any variation in these genes, particularly functional variation, will be essential to maintain both in the wild and in captivity. Targeted assays are now being developed to monitor genetic diversity in these genes both in the wild and captivity [45, 46]. With further research into the Tasmanian devil immune system and its response to DFTD, development of a vaccine or treatment for DFTD may be possible in the near future.

## Methods

### Sample collection, RNA extraction and cDNA library preparation

Tasmanian devil tissue samples were collected under Animal Ethics permits DPIPWE AEC No. 21/2007-08. Spleen and lymph node RNA was extracted from frozen tissue samples (*n* = 1 per tissue) using QIAGEN RNeasy Plus Micro Kit. The concentration of extracted RNA was measured on a NanoDrop (spleen 54 ng/ul, A260/A280 2.00; lymph node 32 ng/ul, A260/A280 1.84) and the quality was checked by visualising on a denaturing TAE gel. Double-stranded cDNA libraries were synthesized using Evrogen MINT cDNA synthesis kit, normalised with Evrogen TRIMMER cDNA normalization kit, and amplified using Clontech Advantage 2 PCR kit.

### Transcriptome sequencing and assembly

Each cDNA library was sequenced on a Roche 454 sequencer using manufacturer’s protocols. For the spleen and lymph node libraries 528,254 and 488,351 reads were obtained respectively. Reads were assembled using Newbler which generated 13,314 and 7544 contigs with an average base length of 869 and 794, (500 bp cutoff) for the spleen and lymph node respectively. The average coverage of contigs longer than 100 nt was 16.2X and 49.4X for spleen and lymph node respectively. Reads were submitted to the European Nucleotide Archive (spleen; [ERA:ERS624952], lymph node [ERA:ERS624953]).

### Gene identification

Tasmanian devil immune genes were identified with a variety of approaches in the two Tasmanian devil genomes (DEVIL7.0, [GenBank:GCA_000189315.1], [12]; [GenBank:GCA_000219685.1], [11]) as well the available Tasmanian devil transcriptomes; tumour and testis ([SRA:SRX015790], [SRA: SRX015793], [28]), milk ([SRA: SRX862745]), and spleen and lymph node. The devil reference genome [12] was the genome primarily used in this study as it is available on Ensembl and has better coverage and higher scaffold lengths. The second genome assembly [10] was used as a supplement when entire gene sequences could not been found in the former. Genes were identified in the Ensembl annotation (Ensembl release 76) of the Tasmanian devil genome where well annotated by Ensembl’s automatic annotation. Unannotated genes, or genes for which the Ensembl annotation was poor or partial, were identified by TBLASTN [47] searches to the Tasmanian devil genome using the relevant sequence in opossum or wallaby from the immunome database for marsupials and monotremes (IDMM; http://hp580.angis.org.au/tagbase/gutentag; [48]), or the relevant human sequences from UniProt when not available in marsupials. In addition, HMMER [49] searches were used to identify genes in families likely to have expansions. To do this, profile hidden Markov models (HMM) were constructed using relevant family members in opossum, human, mouse and chicken. The 6-frame translation of the Tasmanian devil genome was then searched using the constructed profile HMM using HMMER v3.0. This method was used for CXC and CCL chemokines, Type I interferons, *IFNK*, *TNFSF18*, *CSF2* and *OSM*.

For genes not identified by the above methods, TBLASTN (E value cut off of 0.1) searches were performed using opossum or wallaby sequences obtained from IDMM, against the available Tasmanian devil transcriptomes, and the best hits were used as queries to BLAST the Tasmanian devil genome to determine their genomic location. When none of the above methods were successful in identifying orthologs, synteny searches were performed whereby flanking genes of the opossum ortholog were identified. The position of these flanking genes was determined in the Tasmanian devil genome (either through the Ensembl annotation of appropriate BLAST searches) and gene prediction performed using FGENESH+ [50] in the sequence spanning the flanking genes using the opossum ortholog as an input.

### Gene analysis

To confirm their identity, predicted proteins were used as queries in BLASTP (E value cut off of 10) against SWISSPROT. Additionally, identity was confirmed through sequence alignment to orthologs, conservation of protein domains, presence of conserved and characteristic protein features and gene structure comparison. Predicted Tasmanian devil sequences were used to search the available Tasmanian devil transcriptomes using TBLASTX. Alignments were produced in BioEdit [51] using the ClustalW algorithm [52]. Sequence identity was calculated using BioEdit where full sequence orthologs were available for either opossum or human. For phylogenetic tree construction protein sequences were aligned using MUSCLE [53] through the software package MEGA6 [54], using default parameters. Only full-length Tasmanian devil sequences were included in phylogenies. Phylogenetic trees were constructed using the maximum-likelihood method and the Jones-Thorton-Taylor (JTT) model [55], and evaluation through 1000 bootstrap resamplings in MEGA6. Accession numbers of sequences used in these phylogenies can be found in Additional file 1 with the exception of opossum sequences for Figs. 2, 3, 4 and Additional file 3 which were obtained from IDMM (http://hp580.angis.org.au/tagbase/gutentag/).

## Availability of supporting data

The data sets supporting the results of the article are available in the [European Nucleotide Archive] repository; spleen [ERA:ERS624952] and lymph [ERA:ERS624953], and the Immunome Database for Monotremes and Marsupials [http://hp580.angis.org.au/tagbase/gutentag/].

## Additional files

Additional file 1:
**Accession numbers for all data used in this study. Description of data: Table including accession numbers for the genomes, transcriptomes and sequences used in phylogenies in this study.** (CSV 3 kb) Additional file 2:
**Interleukin sequences identified in the Tasmanian devil genome.** Description of data: Md % = percent amino acid identity to opossum (*Monodelphis domestica*) ortholog, Hs % = percent amino acid identity to *Homo sapiens* ortholog. * indicates a gene which is not a direct ortholog of a eutherian gene, ** = partial Ensembl prediction. (CSV 3 kb) Additional file 3:
**Presence of Tasmanian devil sequences identified in this study in the devil transcriptomes.** (CSV 957 bytes) Additional file 4:
**Alignment of devil, opossum and human IL22 amino acid sequences.** Description of data: Amino acid alignment of devil IL22 and IL22 fragments to opossum and human IL22 sequences. Devil sequences IL22F1-IL22F5 are fragments of IL22 identified in fragmented regions of the devil genome. (TIF 14687 kb) Additional file 5:
**Chemokine sequences identified in the Tasmanian devil genome.** Description of data: Md % = percent amino acid identity to opossum (*Monodelphis domestica*) ortholog, Hs % = percent amino acid identity to *Homo sapiens* ortholog. * indicates a gene which is not a direct ortholog of a eutherian gene, ** = partial Ensembl prediction. (CSV 3 kb) Additional file 6:
**Phylogeny of XC family chemokines from devil, opossum and human.** Description of data: XC family chemokines phylogeny from devil, opossum and human amino acid sequences. Maximum likelihood tree with 1000 bootstrap resamplings. Bootstrap value greater than 50 % only are displayed. Devil CCL17 is used as an outgroup. Devil, opossum and human sequences are indicated by red, pink and blue dots respectively. (TIF 1658 kb) Additional file 7:
**Interferon sequences identified in the Tasmanian devil genome.** Description of data: Md % = percent amino acid identity to opossum (*Monodelphis domestica*) ortholog, Hs % = percent amino acid identity to *Homo sapiens* ortholog. * indicates a gene which is not a direct ortholog of a eutherian gene, ** = partial Ensembl prediction. (CSV 564 bytes) Additional file 8:
**TNF family sequences identified in the devil genome.** Description of data: Md % = percent amino acid identity to opossum (*Monodelphis domestica*) ortholog, Hs % = percent amino acid identity to *Homo sapiens* ortholog. * indicates a gene which is not a direct ortholog of a eutherian gene, ** = partial Ensembl prediction. (CSV 1 kb) Additional file 9:
**Additional cytokine sequences identified in the Tasmanian devil genome.** Description of data: Md % = percent amino acid identity to opossum (*Monodelphis domestica*) ortholog, Hs % = percent amino acid identity to *Homo sapiens* ortholog. * = partial Ensembl prediction. (CSV 1 kb) Additional file 10:
**Immunoglobulin heavy and light chain constant regions identified in the Tasmanian devil genome.** Description of data: Md % = percent amino acid identity to opossum (*Monodelphis domestica*) ortholog, Hs % = percent amino acid identity to *Homo sapiens* ortholog. * = partial Ensembl prediction. (CSV 599 bytes) Additional file 11:
**TCR constant regions identified in the Tasmanian devil genome.** Description of data: Md % = percent amino acid identity to opossum (*Monodelphis domestica*) ortholog, Hs % = percent amino acid identity to *Homo sapiens* ortholog. * = partial Ensembl prediction. (CSV 864 bytes)

## Abbreviations

DFTD: Devil facial tumour disease
IUCN: International union for conservation of nature
MHC: Major histocompatibility complex
TLR: Toll-like receptor
TNF: Tumour necrosis factor
IFN: Interferon
TGF: Transforming growth factor
CSF: Colony stimulating factor
TCR: T cell receptors
Ig: Immunoglobulin
HMM: Hidden markov model
IL: Interleukin
TNFSF: Tumour necrosis factor super family
FASLG: Fas ligand
CNTF: Ciliary neurotrophic factor
OSM: Oncostatin M
