# Supplementary figures and images for: Identification and analysis of divergent immune gene families within the Tasmanian devil genome
Source: BMC Genomics. 2015 Nov 26;16:1017. doi: 10.1186/s12864-015-2206-9 (PMC4662006; doi:10.1186/s12864-015-2206-9)

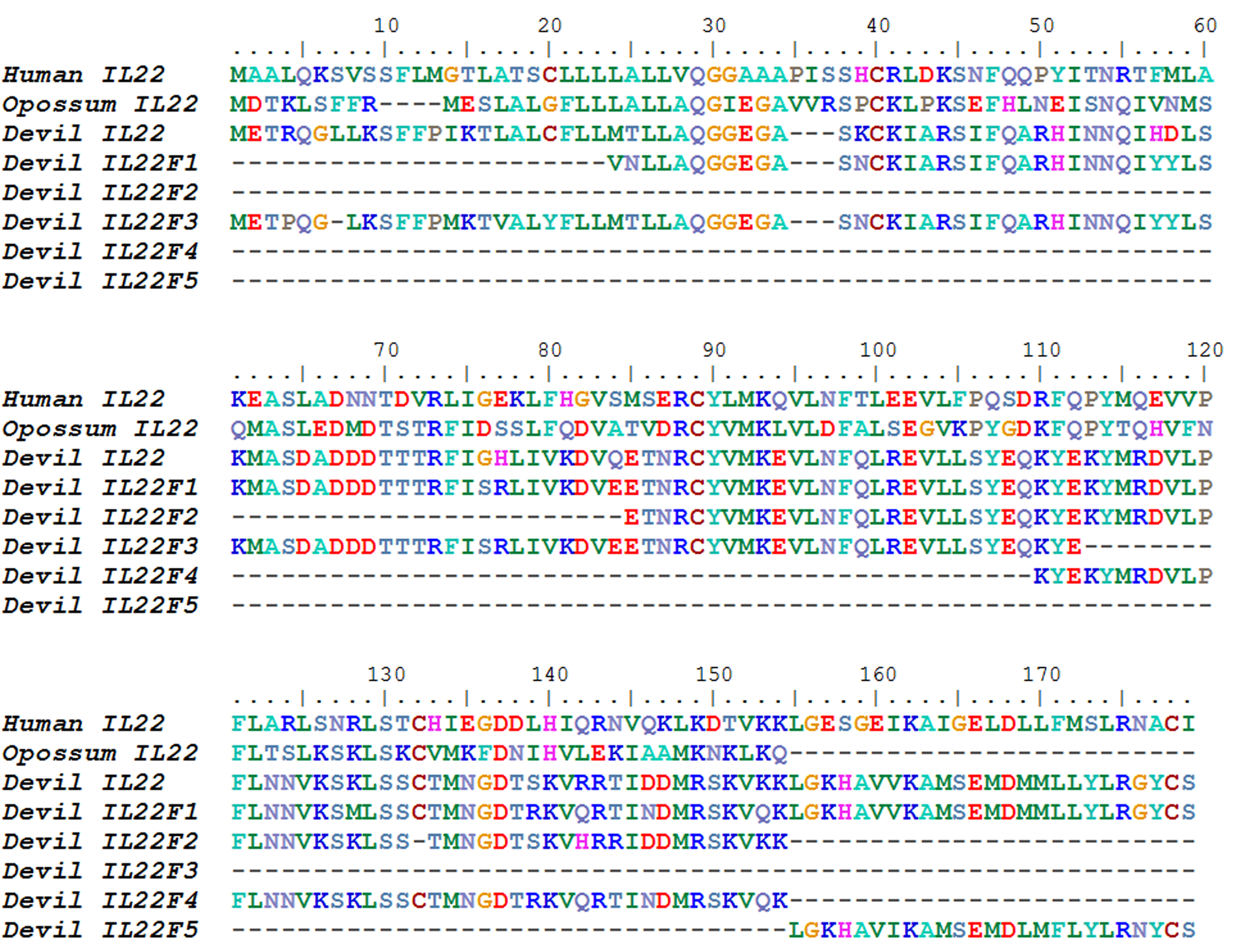

Supplement: Additional file 4: — Alignment of devil, opossum and human IL22 amino acid sequences. Description of data: Amino acid alignment of devil IL22 and IL22 fragments to opossum and human IL22 sequences. Devil sequences IL22F1-IL22F5 are fragments of IL22 identified in fragmented regions of the devil genome. (TIF 14687 kb) [file 12864_2015_2206_MOESM4_ESM.tif]

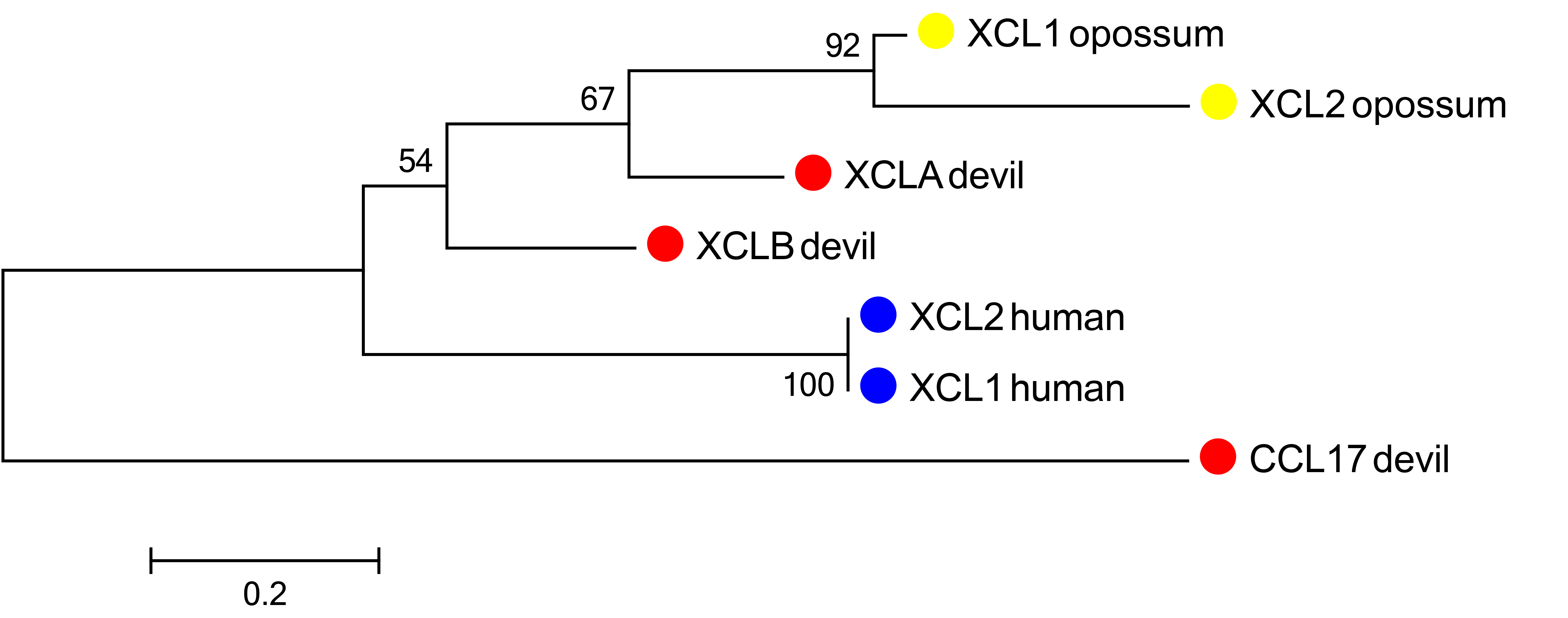

Supplement: Additional file 6: — Phylogeny of XC family chemokines from devil, opossum and human. Description of data: XC family chemokines phylogeny from devil, opossum and human amino acid sequences. Maximum likelihood tree with 1000 bootstrap resamplings. Bootstrap value greater than 50 % only are displayed. Devil CCL17 is used as an outgroup. Devil, opossum and human sequences are indicated by red, pink and blue dots respectively. (TIF 1658 kb) [file 12864_2015_2206_MOESM6_ESM.tif]
